# Association between *MC1R* gene and coat color segregation in Shanxia long black pig and Lulai black pig

**DOI:** 10.1186/s12863-023-01161-2

**Published:** 2023-11-30

**Authors:** Hao Zheng, San-ya Xiong, Shi-jun Xiao, Ze-kai Zhang, Jin-min Tu, Deng-shuai Cui, Nai-biao Yu, Zhi-yong Huang, Long-yun Li, Yuan-mei Guo

**Affiliations:** 1 National Key Laboratory for Swine Genetic Improvement and Germplasm Innovation, Ministry of Science and Technology of China, Jiangxi Agricultural University, Nanchang, Jiangxi 330045 China; 2 The College of Life Science, Nanchang Normal University, Nanchang, Jiangxi 330045 China; 3 Jiangxi Shanxia Huaxi Pig Breeding Company Limited, Ganzhou, Jiangxi 341000 China

**Keywords:** Shanxia long black pig, Lulai black pig, Coat color, GWAS, Causative gene, *MC1R*

## Abstract

**Background:**

Coat color, as a distinct phenotypic characteristic of pigs, is often subject to preference and selection, such as in the breeding process of new breed. Shanxia long black pig was derived from an intercross between Berkshire boars and Licha black pig sows, and it was bred as a paternal strain with high-quality meat and black coat color. Although the coat color was black in the F_1_ generation of the intercross, it segregated in the subsequent generations. This study aims to decode the genetic basis of coat color segregation and develop a method to distinct black pigs from the spotted in Shanxia long black pig.

**Results:**

Only a QTL was mapped at the proximal end of chromosome 6, and *MC1R* gene was picked out as functional candidate gene. A total of 11 polymorphic loci were identified in *MC1R* gene, and only the c.67_68insCC variant was co-segregating with coat color. This locus isn’t recognized by any restriction endonuclease, so it can’t be genotyped by PCR-RFLP. The c.370G > A polymorphic locus was also significantly associated with coat color, and has been in tightly linkage disequilibrium with the c.67_68insCC. Furthermore, it is recognized by *BspHI*. Therefore, a PCR-RFLP method was set up to genotype this locus. Besides the 175 sequenced individuals, another more 1,391 pigs were genotyped with PCR-RFLP, and all of pigs with GG (one band) were black.

**Conclusion:**

*MC1R* gene (c.67_68insCC) is the causative gene (mutation) for the coat color segregation, and the PCR-RFLP of c.370G > A could be used in the breeding program of Shanxia long black pig.

**Supplementary Information:**

The online version contains supplementary material available at 10.1186/s12863-023-01161-2.

## Background

In the past decade, the demand for high-quality pork has increased dramatically in China. Although Chinese indigenous pig breeds can meet this need, they aren’t suitable to be commercialized because of their slow growth and over fatness. To improve the growth and lean rate, tens of maternal strains, but no paternal strain has been bred through intercrossing a Chinese indigenous breed to a commercial breed until now. Shanxia long black pig was derived from an intercross between Berkshire boars and Licha black pig sows, and it was bred as a paternal strain with high-quality meat and black coat color [1]. In the F_1_ of the intercross, almost all of the pigs were black, but coat color was segregating in the F_2_ generation. About 75% pigs were black or black with white socks, and the rest were spotted. This indicates that black and black with white socks are dominant to spotted.

Although each breed has its characteristic coat color, among which white, black, red and spotted are more popular, the coat color of pig is mainly divided into 7 types [2]. White is the major coat color in commercial pig breeds over the world, in fact there are other kinds of coat color, such as unexpected spotted [3, 4].

In Chinese pig market, it is generally believed that black pigs have higher meat quality than white pigs, therefore consumers are favorer black pigs than white pigs. To fix the dominant black, phenotype selection is inefficient for limitations in culling the recessive spotted completely. On the contrary, gene selection is efficient to fix the favor allele and eliminate the adverse allele [5]. The essential prerequisite of gene selection is identifying the causative gene of the trait [6]. Coat color is a qualitative, but it is a complicate trait. For example, white coat of Rongchang pig is not caused by the mutation of *KIT* gene like Landrace and Yorkshire [7]. By now, GWAS is an ordinary method to map the QTL of a trait, and then combines with fine mapping and positional candidate gene approaches to identify the causative gene. Although the study of coat color is traced back to the early 20th century in pigs [8], only hands of the causative genes have been identified so far, such as *KIT*, *MC1R*, *ASIP*, *TYRP1*, and *EDNRB* [9–12].

For piebald phenotype, Kjias proposed that a frame shift mutation CC insertion of *MC1R* caused black spots in pigs [13]. In 2017, Wu et al. took *MC1R* and *ASIP* as candidate genes for black spots in Qingyu pigs, and CC insertion of *MC1R* associated with coat color separation in Qingyu pig [4]. *MC1R* gene locates in the short arm of chromosome 6 of porcine and has six alleles: E^+^ corresponds to the coat color of wild boar; E^D1^ (p.Val95Met; p.Leu102Pro) is the major allele for the black coat color in Chinese indigenous pigs, Licha black pig and Lulai black pig belong to or derive from Chinese indigenous pig breed, so E^D1^ is the major allele in these two breeds; E^D2^ (p.Asp124Asn) is the major allele of western Hampshire pigs; E^P^ (p.Asp124Asn; c.67_68insCC) relates to the coat color of Pietrain and Berkshire; e (p.Ala164Val; p.Ala243Thr) determines Duroc red or yellow coat; e^IB^ (c.67_68insCC) belongs to Iberian variety [14]. E^D1^ is completely dominant to E^P^ and e, and E^P^ is incompletely dominant to e. In 2001, Deng et al. found a new allele E^S^ in Shengxian spotted pigs, but its effect on coat color was unknown [15]. In addition to the identified causative genes such as *MC1R* and *KIT*, coat color is also regulated by some other genes [16].

To identify the causative mutation for coat color segregation in Shanxia long black pig and Lulai black pig, a GWAS was performed to mapping the QTL for coat color, and functional candidate gene was selected in the QTL region. Polymorphic loci of the candidate gene were searched with Sanger sequencing, and the underlying causative mutation was chosen. More pigs with coat color were genotyped with PCR-RFLP, and the causative mutation was verified according to the co-segregation between the variant and coat color. After having been testified, the causative mutation has been applied for gene selection in Shanxia long black pig.

## Results

### Coat colors in each population

**Fig. 1:**
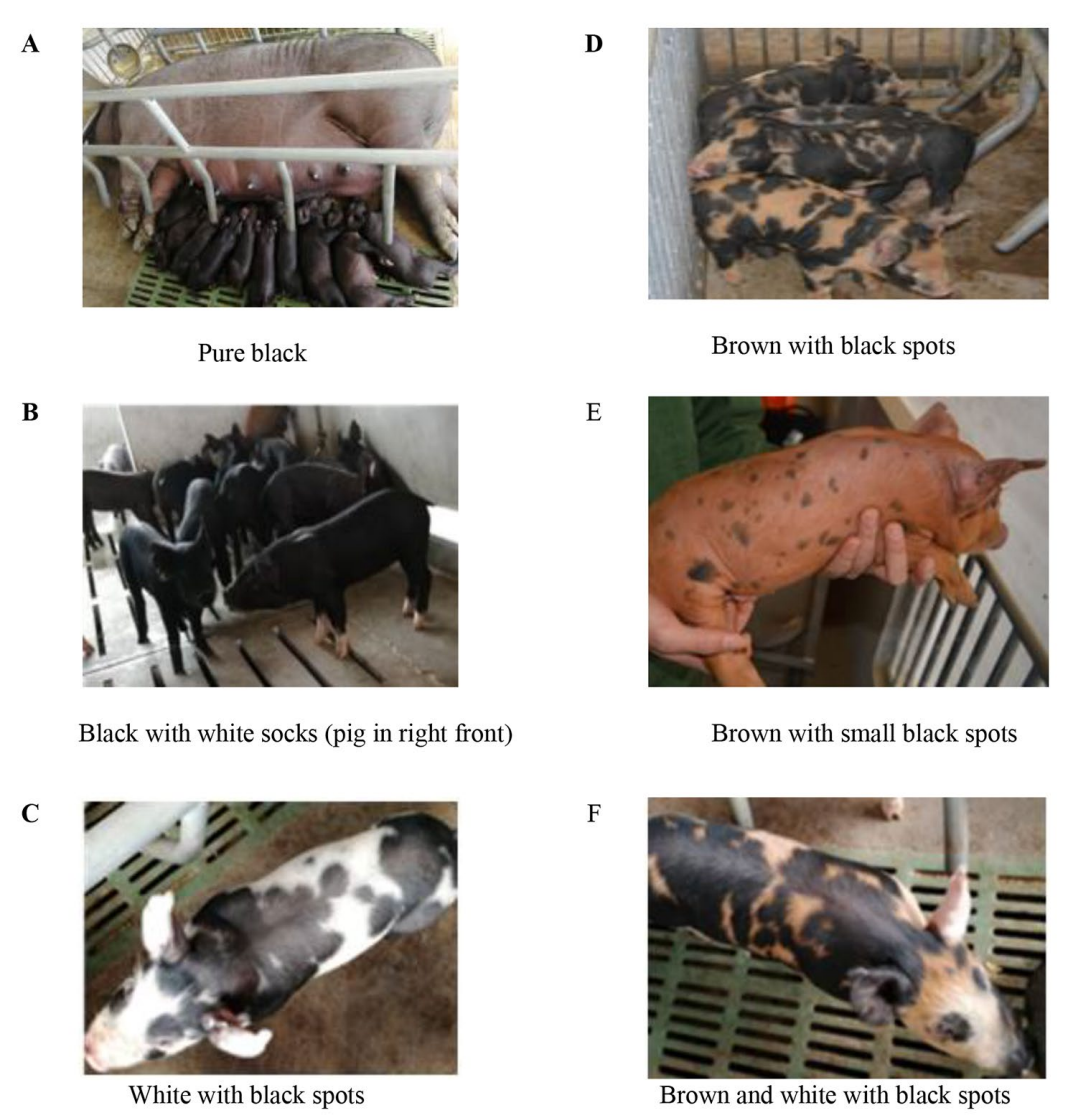
Coat color in an intercross between Berkshire boars and Licha black pig sows

There were six kinds of coat colors observed in the two populations (Fig. 1). In Chinese pig market, consumers like black pigs more than spotted pigs, and they accept black pigs with white socks as same as black pigs. Therefore, the coat color was divided into two types: one was black including black and black with white socks, the other was spotted including the remaining four kinds. The coat colors of Licha black pig and Berkshire were mainly black and black with six whites (white socks, snout and tip of tail), respectively. Lulai black pig and BL_1_, namely first generation of the cross between Berkshire boars and Licha black pig sows (BL), were black except few spotted pigs. In BL_2_ (second generation of BL) and BL_3_ (third generation of BL), most pigs were black, but some pigs were spotted, and there were more spotted pigs in BL_2_ than in BL_3_. Table 1 listed the number of pigs for each coat color in each population. All the 11,263 Licha black pigs were black except 3 spotted pigs (0.03%), so the coat color was almost fixed in Licha black pig. More than 90% Berkshires were black with six whites (belonging to spotted pigs), and the rest (9.09%) were black with white socks. All the 24,236 BL_1_ were black except 51 spotted pigs (0.21%). In BL_2_ and BL_3_, the coat color was segregated. For the 40,556 BL_2_, about three quarters of them (30,316, 74.75%) were black or black with white socks, and the rest (10,240, 25.25%) were spotted. The coat color was selected in BL_2_ through phenotype selection, only the black pigs were kept, therefore, the proportion of spotted pigs was dropped from 25.25% in BL_2_ to 9.87% in BL_3_.

**Table 1:** Numbers of black and spotted pigs in each herd

Herd	Black or black with white socks		Spotted		Total
	*n*	Percentage, %	*n*	Percentage, %	
Licha black pig	11,263	99.97	3	0.03	11,266
Berkshire	29	9.09	290	90.91	319
F_1_ of Berkshire × Licha black pig	24,236	99.79	51	0.21	24,287
F_2_ of Berkshire × Licha black pig	30,316	74.75	10,240	25.25	40,556
F_3_ of Berkshire × Licha black pig	4931	90.13	540	9.87	5471
Total	70,775	86.42	11,124	13.58	81,899

Only two litters of Lulai black pig, which were from a boar mating to 2 full-sib sows, were appearing coat color segregation. A total of 20 piglets were produced in the two letters, including 15 black and 5 spotted piglets.

### The inheritance model of coat color

Chi-square tests showed the ratios of black pigs to spotted pigs did not deviate from the Mendelian segregation ratio of 3:1 in BL_2_ (*χ*^2^ = 1.3415, *P* = 0.2468) and Lulai black pig (*χ*^2^ = 0, *P* = 1.0). This indicated that a single gene on an autosome caused the segregation of coat color, and black was completely dominant to spotted.

### GWAS results of coat color

**Fig. 2:**
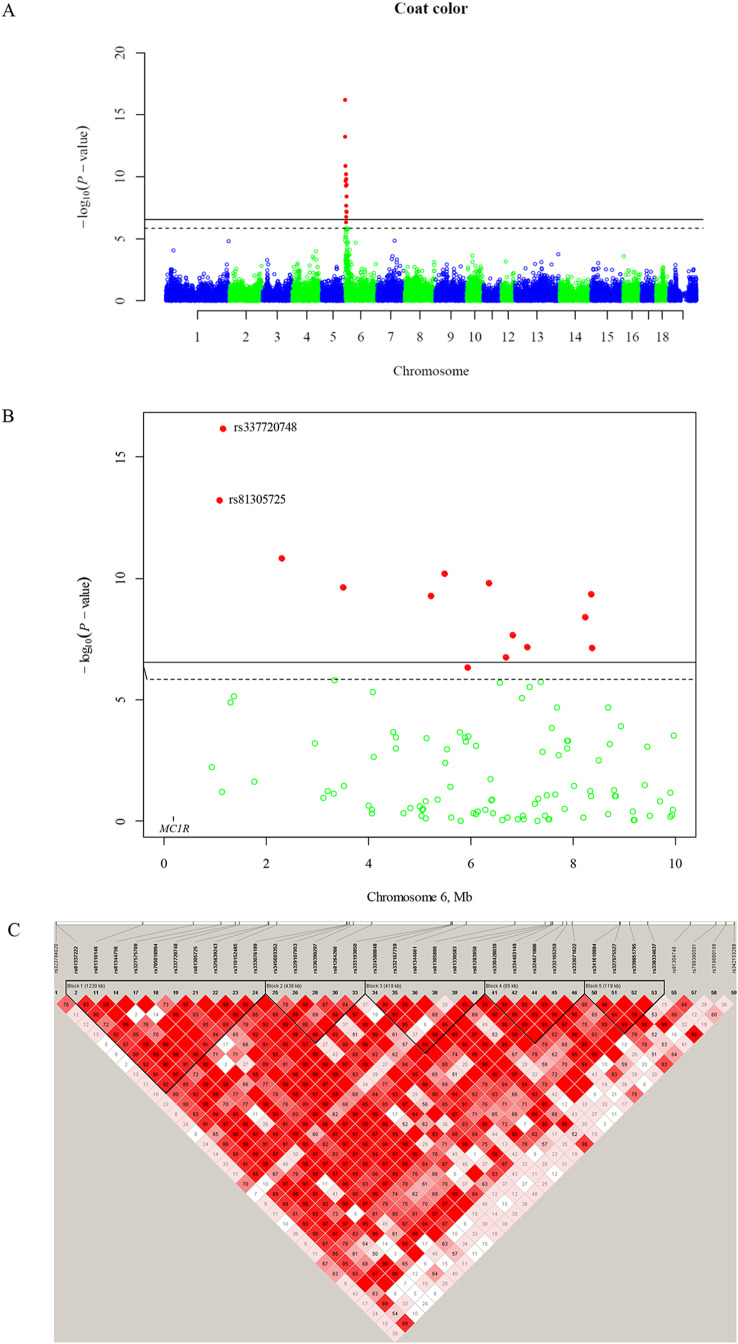
Manhattan plot for coat color and linkage disequilibrium in the top SNP region. (**A**) Genome-wide Manhattan plot; (**B**) Manhattan plot in the top SNP region; (**C**) Linkage disequilibrium in the top SNP region. The *x* axis indicates the position and chromosome of each SNP, and the *y* axis is the negative common logarithm of the *P* value. The solid and dashed horizontal lines show the 1% and 5% genome-wide significant levels, respectively

**Fig. 3:**
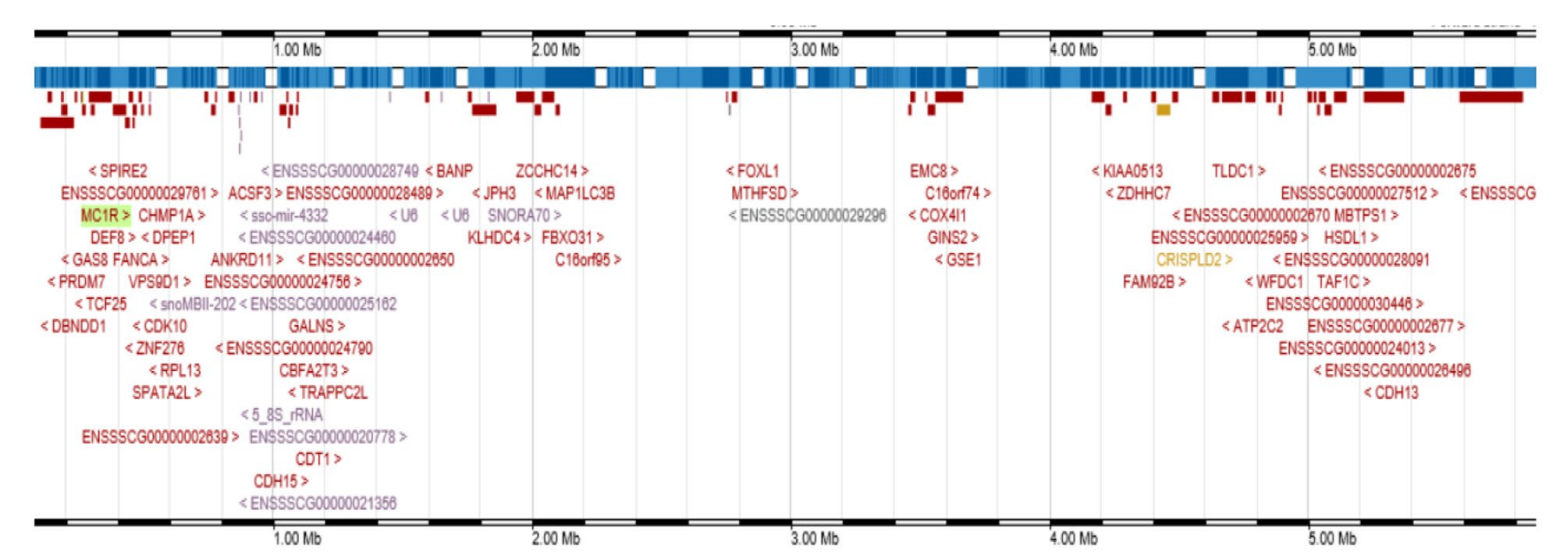
Annotated genes at the end of the short arm of chromosome 6

A total of 34,898 SNPs and 1,156 individuals passed the quality control and retained for subsequent analysis. GWAS identified 14 SNPs in association with coat color at genome-wide significant level on chromosome 6 (Fig. 2, S1 and S2, and Table 2). All the 14 SNPs were mapped in the region from 0.787 to 8.321 Mb, and *MC1R* gene that regulates the process of melanin synthesis in melanocytes is about 500 kb upstream to the top SNP (Fig. 3). Although there were 13 SNPs within 500 kb to *MC1R* gene in the Chinese Chip-1 PorcineSNP50 BeadChip, only one SNP passed the quality control and used to perform association analysis with coat color, but it was not significant (*P* = 0.0058) at genome-wide significant level (Table S1). The other 12 SNPs were discarded because of low polymorphism (MAF < 0.05) or seriously deviating from Hardy - Weinberg equilibrium (*p* < 0.00001). Therefore, no SNP associated with coat color within 500 kb to *MC1R* gene. Considering the significant role *MC1R* plays in the diversity of pig coat color, we still consider it as a strong candidate gene for coat color segregation here.

**Table 2:** SNPs associated with coat color at genome-wide significant level

SNP	Chromosome	Position, bp	Allele	Additive effect ± SE.	*P* value
rs337720748	6	787,448	A	0.101 ± 0.0105	6.48E-17
rs81305725	6	909,464	A	0.085 ± 0.0099	5.93E-14
rs324671806	6	2,156,953	A	0.099 ± 0.0127	1.42E-11
rs81238557	6	5,154,694	A	0.086 ± 0.0114	6.33E-11
rs346300681	6	5,821,382	G	0.055 ± 0.0074	1.57E-10
rs81334396	6	3,193,701	G	0.064 ± 0.0087	2.24E-10
rs81346632	6	8,245,682	C	0.077 ± 0.0107	4.37E-10
rs81307446	6	4,817,058	A	0.054 ± 0.0076	5.32E-10
rs81284048	6	8,219,229	A	0.052 ± 0.0077	4.03E-09
rs81393241	6	6,233,415	A	0.048 ± 0.0074	2.12E-08
rs81394823	6	6,587,482	C	0.051 ± 0.0081	6.62E-08
rs81248474	6	8,320,740	A	0.081 ± 0.0131	7.31E-08
rs81394715	6	6,100,613	G	0.062 ± 0.0103	1.71E-07
rs81388192	6	5,400,895	G	0.044 ± 0.0076	4.53E-07

### Linkage disequilibrium in the top SNP region

In the top SNP (rs337720748) region, there is a linkage disequilibrium (LD) block, which covers a 1230 kb region from 10.727 to 1,241.022 kb (Fig. 2C), and *MC1R* gene is in this LD block. Therefore, we proposed *MC1R* gene as the candidate gene for coat color.

### Polymorphic loci of ***MC1R***

The amplified fragment sizes of PCR for primer 1 and 2 were very close to their expected sizes of 750 and 880 bp according to the electropherogram (Figure S3), so the PCR products were sent to sequence. The sequences of PCR products were blasted to the reference sequences of *MC1R* in the NCBI database (https://blast.ncbi.nlm.nih.gov/Blast.cgi). The percent identity between the sequenced and reference sequences was more than 99%, which indicated that the amplified fragments were the target fragments.

Using the sequences of 175 pigs, polymorphic loci in the coding region of *MC1R* gene were searched by using SeqMan software in DNASTAR package. A total of 10 polymorphic loci were found in the 127 BL pigs, namely, c.51G > A, c.67_68insCC, c.283G > A, c.305T > C, c.363T > C, c.364G > A, c.370G > A, c.491T > C, c.727 A > G and c.729G > A. Besides the 10 polymorphic loci, another polymorphic locus (c.288G > A) was found in the 48 Lulai black pigs. Because the loci of c.491T > C and c.727 A > G were non-polymorphic in Lulai black pig and c.288G > A was non-polymorphic in BL, they were not used in the association analysis.

### Candidate causative polymorphic locus

The association analysis results were shown in Table 3. The c.67_68insCC locus associated with coat color was the most significant, and the *P* values were 2.64E-28 and 5.84E-07 in the two populations, respectively. Only this locus could completely distinguish black pigs (--/-- and --/CC) from spotted pigs (CC/CC) matched the autosomal dominant inheritance completely, so it was most likely the causal mutation for the coat color segregation in the two populations.

**Table 3:** The association between coat color and polymorphic loci of *MC1R* gene

Site	Genotype	Cross between Berkshire and Licha black pig					Lulai black pig		
		Black	Spotted	*χ* ^2^	*df*	*P* value	Black	Spotted	*P* value
	A/A	52	0				31	0	
c.51G > A	A/G	30	0	95.14	2	2.19E-21	12	0	5.84E-07
	G/G	8	37				0	5	
	--/--	60	0				31	0	
c.67_68insCC	CC/--	30	0	127	2	2.64E-28	12	0	5.84E-07
	CC/CC	0	37				0	5	
	A/A	52	0				31	0	
c.283G > A	A/G	30	0	95.14	2	2.19E-21	12	0	5.84E-07
	G/G	8	37				0	5	
	C/C	52	0				31	0	
c.305T > C	C/T	30	0	95.14	2	2.19E-21	12	0	5.84E-07
	T/T	8	37				0	5	
	C/C	52	0				31	0	
c.363T > C	C/T	30	0	95.14	2	2.19E-21	12	0	5.84E-07
	T/T	8	37				0	5	
	A/A	1	0				0	0	
c.364G > A	A/G	22	0			0.000289	1	0	1.000
	G/G	67	37				42	5	
	A/A	8	37				0	5	
c.370G > A	A/G	30	0	95.14	2	2.19E-21	12	0	5.84E-07
	G/G	52	0				31	0	
	A/A	52	0				31	0	
c.729G > A	A/G	30	0	95.14	2	2.19E-21	12	0	5.84E-07
	G/G	8	37				0	5	

Note: *P* values without chi-square value and degree of freedom are from Fisher exact test

### The genotyping protocol to distinguish black pigs from spotted pigs

Restriction enzyme site was searched for the c.67_68insCC locus by using Bioxm 2.6 nucleotide sequence analysis software, but no endonuclease recognized the locus, so this locus couldn’t be genotyped through a PCR-RLFP method. The c.370 G > A locus was genotyped instead of the c.67_68insCC locus, because this locus could be recognized and digested by the restriction endonuclease *BspHI*, and its genotype GG was completely associated with black coat color. *BspHI* cut the nucleic acid sequence “-TCATG*A*-” with the mutation allele into two fragments, but couldn’t cut the reference sequence. The locus was in the amplification region of primer 2, and the 880 bp PCR product with mutative allele was cut into a 750 and a 130 bp fragments. Therefore, according to the number of fragments, the genotypes of AA, AG and GG were 2 (130 and 750 bp), 3 (130 bp, 750 and 880 bp) and 1 (880 bp) respectively, and the individuals with GG were homozygotes with black coat color (Figure S4).

### Genotyping results of PCR-RFLP

Besides the 175 sequenced individuals, another more 1,391 pigs were genotyped with PCR-RFLP, and their genotypes and coat colors were listed in Table 4. Result shows that pigs with AA were spotted or black, while all of pigs with GG (one band) were black. Therefore, selecting the GG individuals could cull spotted pigs in Shanxia long black pig and Lulai black pig.

**Table 4:** The association between coat color and genotypes determined by PCR-RFLP

Coat color	Fragment number	Licha black pig (LC)	Berkshire(BS)	BS × LC	Lulai black pig
	1 (GG)	114	0	329	55
Black	2 (AA)	0	0	18	0
	3 (AG)	27	0	581	25
Spotted	2 (AA)	0	9	403	5

## Discussion

In this study, a genome-wide association analysis was performed, and a total of 14 SNPs at the proximal end of SSC6 associated with coat color at genome-wide significant level. *MC1R* gene was picked out as a strong functional candidate gene for coat color in this region, and its polymorphic loci were searched in 175 individuals by Sanger sequencing. A total of 11 polymorphic loci were found, and only the c.67_68insCC polymorphic locus followed the completely autosomal dominant inheritability model for coat color. This locus could completely distinguish black pigs (--/-- and --/CC) from spotted pigs (CC/CC), so it was the causal mutation that led to the segregation of the coat color in the two populations.

The coat color follows a dominant inheritance model in this study, so a dominant model (Figure S2B) is more powerful than an additive model (Figure S2A). Both the additive (Fig. 2 and S2A) and the dominant (Figure S2B) models give the same result, and only identify a QTL at the proximal end of chromosome 6. Considering most GWAS are performed with an additive model, so GWAS with an additive model is performed in this study.

There are 29 protein coding genes within the 500 kb region of the most significant locus, but there is no literature supporting them in association with pig coat color. *MC1R* gene is about 500 kb upstream to the top SNP. Additionally, *MC1R* in association with different kinds of coat colors have been documented in Chinese native breeds, Landrace, Yorkshire, Duroc, Hampshire [17, 18], and other species such as cattle [19], sheep [20], dog [21] and horse [22]. The *MC1R* gene codes melanocortin 1 receptor protein, which regulates the process of melanogenesis in melanocytes [23, 24]. Furthermore, Shanxia long black pigs are derived from Berkshire with six-white-point coat color, which carries the E^P^ allele, including c.67_68insCC and c.370G > A. Spotted coat color in Pietrain and the white coat color in the Bama miniature pig population are both due to c.67_68insCC, leading to premature stop codon at position 56 and loss of function of *MC1R* [13, 25]. While c.67_68insCC was known to be present in the Pietrain genome, and transcripts without CC insertions were found in colored skin, presumably due to persistent 8 C instability [13]. This suggests that the main cause of the pattern in the black pig population is the E^P^ allele inherited from Berkshire. Therefore, *MC1R* was chosen as functional candidate gene for coat color in Shanxia long black pigs.

A total of 11 polymorphic loci have been found, among which 7 loci were also polymorphic in Qingyu pig [4], c.364G > A was polymorphic in wild boar [14], c.491T > C and c.727 A > G were reported in Yunan black pig [26], and only c.288G > A was newly revealed in this study. Although the c.67_68insCC locus is the causative mutation for coat color, it cannot be genotyped with PCR-RLFP without restriction endonuclease recognizing this locus. The c.370G > A locus associated with coat color highly significantly could be cut by restriction endonuclease *BspHI *[13]. Based on the genotyping results of 1,566 pigs, all individuals with GG were black. Therefore, keeping the pigs with GG could eliminate the spotted pigs from the Shanxia long black pig. Choosing pigs with GG of c.370G > A will result in culling some black pigs with --/-- of c.67_68insCC. If there are enough black pigs in the breeding population, like this study, genotyping c.370G > A by PCR-RFLP is more convenient and economic efficient. If there are few black pigs in the candidate population, genotyping c.67_68insCC by TaqMan PCR or mismatch primer PCR is more powerful.

There were only 5 spotted and 80 black pigs in Lulai black pig population, and the small population was not suitable to perform a GWAS alone [27]. According to the results of GWAS and the functional candidate gene approach, *MC1R* gene was selected as the supposed causative gene for coat color in Lulai black pig. After sequencing 48 Lulai black pigs (including 43 black and 5 spotted pigs), 11 polymorphic loci were detected in *MC1R* coding region. Except the c.288G > A and c.364G > A loci, all of them could be used to distinguish black pigs from spotted pigs (Table 3). As same reason as in Shanxia long black pig, the c.370G > A locus was selected to genotype through PCR-RFLP [28]. Another 37 Lulai black pigs with black coat color were genotyped, and all of their genotypes were GG. Taking consider of only 5 spotted pigs genotyped in Lulai black pig, the c.370G > A locus co-segregating with coat color might be caused by accidence. Therefore, it was necessary to genotype more spotted pigs to verify that *MC1R* gene was the causative gene for the segregation of coat color in Lulai black pig.

## Conclusions

*MC1R* gene (c.67_68insCC) is the causative gene (mutation) of coat color segregation, and genotyping c.370G > A by PCR-RFLP could be used in the breeding program of Shanxia long black pig.

## Methods

### Animals and coat color recording

There were two experimental pig populations used in this study, and both were from Jiangxi Shanxia Huaxi Pig Breeding Co., Ltd. (Ganzhou, Jiangxi province, China). One was Shanxia long black pig, including Berkshire (*n* = 319), Licha black pig (*n* = 11,266), and the first (BL_1_, *n* = 24,287), second (BL_2_, *n* = 40,556) and third (BL_3_, *n* = 5,471) generations of an intercross between Berkshire boars and Licha black pig sows (BL). The other was Lulai black pig (*n* = 201), which was bred from a cross between Large White and Laiwu black pig and had been artificially selected for more than 8 generations. Coat color of each pig was recorded at birth after the pig having identification, and the numbers of black and spotted pigs were counted in each population.

### Testing the inheritance model of coat color

Based on the ratio of black pigs to spotted pigs, the autosomal dominant inheritance pattern of coat color was tested using a chi-square test and a Fisher’s exact test with *chisq.test* and *fisher.test* functions of R in BL_2_ and Lulai black pig, respectively.

### Genotyping and genome-wide association analysis

Tissue sample of each experimental pig was collected from the ear and put into an Eppendorf tube filled with 75% ethanol. A phenol-chloroform method was used to extract genomic DNA for each pig from its ear sample, and the quality of DNA was checked using Nanodrop-1000 ultra-micro spectrophotometer. DNA with the ratio of A260 to A280 between 1.8 and 2.0 and the ratio of A260 to A230 greater than 1.9 was diluted to 50 ng/μL.

A total of 1,160 pigs, including 1,123 black and 37 spotted pigs (Table S2), were genotyped for 51,368 SNPs using the Chinese Chip-1 (CC1) PorcineSNP50 BeadChip (Illumina, San Diego, CA, USA) [1]. The SNPs were filtered using the GenABEL package of R according to call rate ≥ 90%, minor allele frequency (MAF) ≥ 5%, and *P* value of a Hardy-Weinberg equilibrium test ≥ 10^− 5^. Individuals with the call rate of SNPs < 90% were discarded.

Black and spotted were set as control and case, respectively. GenABEL package of R was used to perform the genome-wide association analysis with an additive effect model [29]. Coat color is a qualitative trait and almost unaffected by other factors, so no other effects are fitted in the GWAS model. To remove the population stratification effect, a polygenic addictive effect following *N*(**0**, ***G***$\sigma_u^2$) was fitted in the model as a random effect, where ***G*** was the genomic kinship matrix and $\sigma_u^2$ was the additive variance [30]. The genomic control was used to correct the remaining population stratification effect after having been adjusted by polygenic addictive effect.

Coat color was inferred to following a dominant inheritance model in this study, so an additive and dominant model was more powerful than an additive model. A GWAS with an additive and dominant model was performed using *qtscore* function of GenABEL package, and population was included in the model to remove the population stratification effect.

Bonferroni correction was used to determine the statistical significance of GWAS, and the threshold *P* values for 5% and 1% genome-wide significant levels were equal to 0.05 and 0.01 divided by the number of qualified SNPs, respectively.

### Linkage disequilibrium analysis

Haplotypes of target regions were inferred by PLINK [31], and linkage disequilibrium (LD) blocks were constructed by Haploview4.2 under the default parameters [32].

### Searching ***MC1R*** polymorphic sites

Genome-wide association analysis of coat color only identified one genome-wide QTL at the proximal end of chromosome 6 (SSC6), and *MC1R* was a strong functional candidate gene in this region. To test whether *MC1R* led the coat color segregation in BL and Lulai black pig, a total of 175 pigs were randomly selected and sent to sequence (Table S2), including 9 Berkshire (six-white-point coat color), 47 Licha black pig (black), 7 BL_1_ (black), 52 BL_2_ (17 black, 25 spotted and 10 black with white socks), 12 BL_3_ (4 black, 3 spotted and 5 black with white socks) and 48 Lulai black pig (43 black and 5 spotted).

The DNA reference sequence of the region from 180,725 to 182,687 (*sscrofa*11.1) on SSC6 was downloaded from the ensemble website (http://asia.ensembl.org/index.html), and the PCR primers were designed by primer 3.0 online (http://primer3.ut.ee/). Two pairs of primers were used to amplify the open reading frame of *MC1R*, and the expected fragment sizes of PCR products were 750 and 880 bp, respectively. The forward primes were (5’-TCTCCAGGGAAGACTTGGTG-3’) and (5’-GCACTCGCCCATGTACTACT-3’), and the backward primes were (5’-GGCAGGGAAGGTGTTTGTTA-3’) and (5’-GACTGGCCTCTGTCCCTTG-3’) for the 2 primers. *MC1R* was amplified by PCR with a 25μL reaction system, which contained 2μL genomic DNA, 1μL forward and 1μL backward primes, 12.5μL 2×Taq Master Mix (Vazyme, Nanjing) and 8.5μL ddH_2_O. The amplification cycling of primer 1 was 94 °C for 5 min, 94 °C for 30s, 56.5 °C for 30s, 72 °C for 50s, 34 cycles, 72 °C for 10 min, and of primer 2 was 94 °C for 5 min, 94 °C for 30s, 56 °C for 30s, 72 °C for 1 min, 34 cycles, 72 °C for 10 min.

PCR amplified products were checked through 1.5% agarose gel electrophoresis, and the qualified PCR products were sent to a biological company for Sanger sequencing (Qingke, Hunan, China). To conform whether the amplified region was the target region, the sequencings of PCR products were blasted to the pig reference sequences of *MC1R* using Nucleotide BLAST on NCBI website (https://blast.ncbi.nlm.nih.gov/Blast.cgi). After the sequences having been verified, SeqMan in DNASTAR software packages (Madison, WI, USA) was employed to search for polymorphic loci in *MC1R* and output the genotypes of polymorphic loci for sequenced individuals.

### Screening out the causative polymorphic locus and establishing its genotyping protocol

After having got the genotypes of *MC1R* polymorphic sites, the candidate causative polymorphic locus, which could distinguish black pigs from spotted pigs, were screened out. Sequencing could genotype all polymorphic sites at once time, but it was costly and time-consuming. An economical and time-saving genotyping protocol was necessary to establish for large-scale genotyping [33]. Bioxm 2.6, which was developed by Ji Huang from Nanjing Agricultural University, was used to search a polymorphic locus for PCR-RFLP detection.

According to the amplification reaction system and reaction conditions of the primer 2 described above, the target sequence was amplified. The qualified PCR products were digested with *BspHI* using a 25μL enzyme digestion system (3μL PCR product, 0.5μL 10,000 units/ml *BspHI*, 2.5μL 10×Cut Smart Buffer and 19μL nuclease-free ddH_2_O) incubating for 40 min under 37 °C. The digested PCR products were denatured and electrophoresed on a 1.5% agarose gel at 200 V for 25 min, and then were visualized under UV light after ethidium-bromide staining. The genotype of each individual was determined according to the number and size of fragments. A total of 175 sequenced pigs, including 127 BL and 48 Lulai black pigs, were genotyped to testify the coordination between the genotypes from sequencing and PCR-RFLP. Another 1,354 BL and 37 Lulai black pigs were genotyped using PCR-RFLP. Chi-square test (*chisq.test* in R) and Fisher’s exact test (*fisher.test* in R) were used to test the association between SNP and coat color in BL and Lulai black pig, respectively.

### Electronic supplementary material

Below is the link to the electronic supplementary material.

Supplementary Material 1

Supplementary Material 2

Supplementary Material 3

Supplementary Material 4

Supplementary Material 5

Supplementary Material 6

## Data Availability

The *MC1R* sequence data generated and analyzed during the current study are available in the National Center of Biotechnology Information (https://www.ncbi.nlm.nih.gov) with accession number OL958548, OL958549 and OL958550.

## Abbreviations

*ASIP*: Agouti signaling protein
BL: An intercross between Berkshire boars and Licha black sows
*EDNRB*: Endothelin receptor type B
GWAS: Genome-wide association study
*KIT*: Receptor tyrosine kinase
MAF: Minor allele frequency
*MC1R*: Melanocortin 1 receptor
PCR-RFLP: Polymerase Chain Reaction-restriction fragment length polymorphism
PCR: Polymerase Chain Reaction
QTL: Quantitative trait locus
SNP: Single nucleotide polymorphism
*TYRP1*: Tyrosinase related protein 1
