# Supplementary material for: Association between MC1R gene and coat color segregation in Shanxia long black pig and Lulai black pig
Source: BMC Genom Data. 2023 Nov 30;24:74. doi: 10.1186/s12863-023-01161-2 (PMC10691012; doi:10.1186/s12863-023-01161-2)
Supplement: Supplementary file 1 — Supplementary Material 1 [file 12863_2023_1161_MOESM1_ESM.docx]

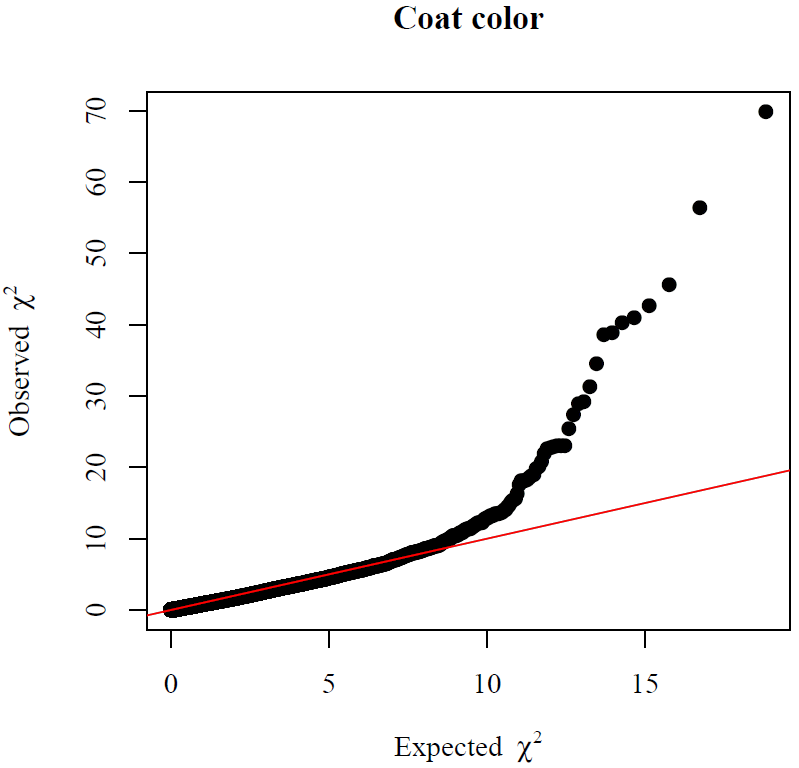


**Figure S1 QQ-plot of GWAS for coat color**. The *x* axis is expected chi-squared values following the chi-squared distribution with 1 degree of freedom, and the *y* axis is observed chi-squared values.
