# Supplementary material for: Association between MC1R gene and coat color segregation in Shanxia long black pig and Lulai black pig
Source: BMC Genom Data. 2023 Nov 30;24:74. doi: 10.1186/s12863-023-01161-2 (PMC10691012; doi:10.1186/s12863-023-01161-2)
Supplement: Supplementary file 2 — Supplementary Material 2 [file 12863_2023_1161_MOESM2_ESM.docx]

| A | 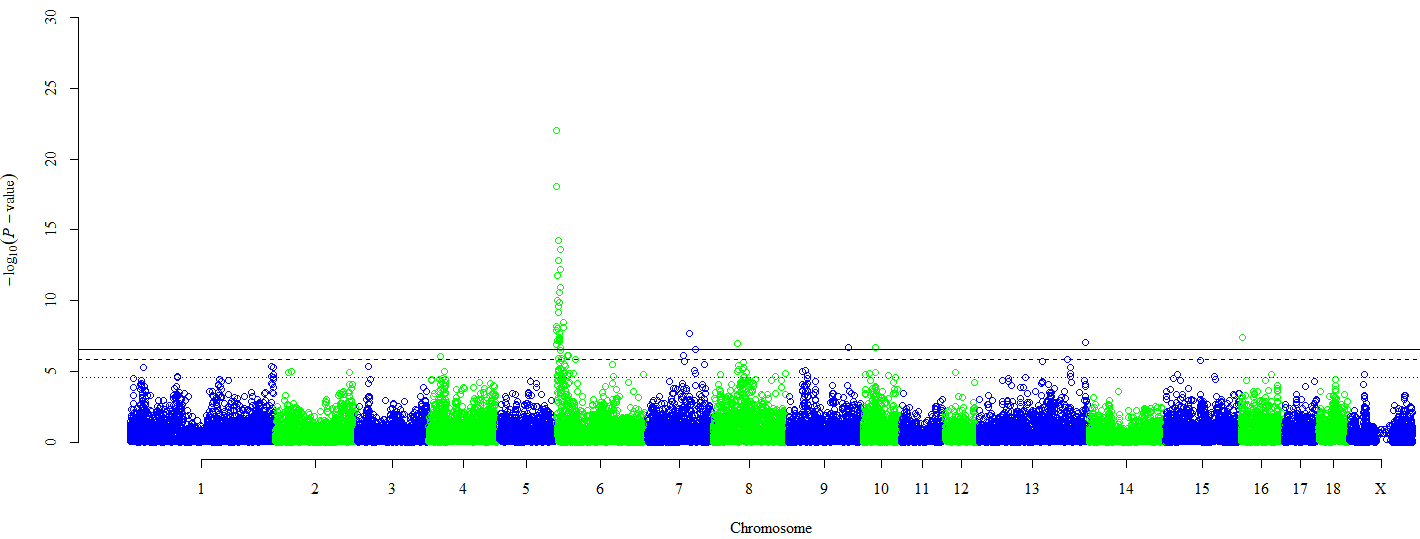 |
| --- | --- |
| B | 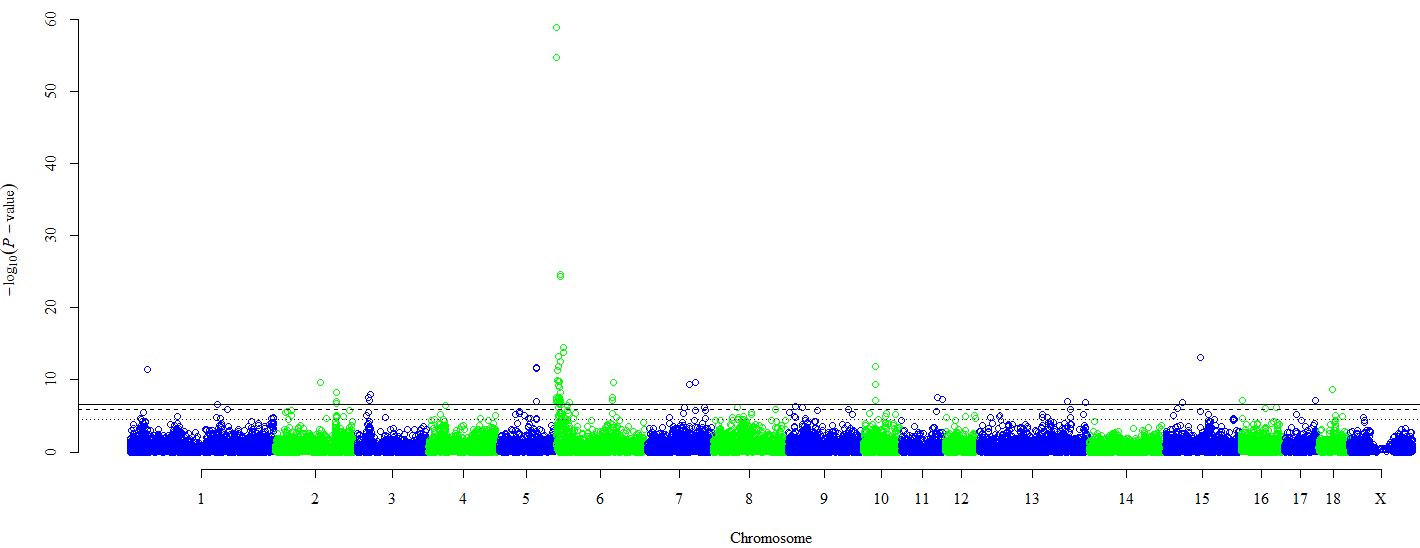 |

**Figure S2** **Manhattan plot for coat color.** A. Additive model; B. Dominant model. The *x* axis indicates the position and chromosome of each SNP, and the *y* axis is the negative common logarithm of the *P* value without being corrected by genomic control. The solid, dashed and dotted horizontal lines show the 1% and 5% genome-wide and suggestive significant levels, respectively.
