# Supplementary material for: Association between MC1R gene and coat color segregation in Shanxia long black pig and Lulai black pig
Source: BMC Genom Data. 2023 Nov 30;24:74. doi: 10.1186/s12863-023-01161-2 (PMC10691012; doi:10.1186/s12863-023-01161-2)
Supplement: Supplementary file 3 — Supplementary Material 3 [file 12863_2023_1161_MOESM3_ESM.docx]

Table S1 The quality-control results of SNPs within 500 kb to *MC1R* gene

| SNP | Position, bp | HW *P* value | Alleles | Minor allele frequency |
| --- | --- | --- | --- | --- |
| rs323784020 | 10706 | 1.64E-11 | A:G | 0.426 |
| rs81337222 | 10727 | 5.78E-30 | C:A | 0.242 |
| rs81328160 | 128223 | 1 | G:A | 0.009 |
| rs333345737 | 142567 | 1 | -:C | 0 |
| rs700918924 | 298090 | 1 | -:G | 0 |
| rs81330557 | 343293 | 1 | -:A | 0 |
| rs81310146 | 378156 | 3.37E-17 | G:A | 0.348 |
| rs81326398 | 451496 | 1 | A:G | 0.001 |
| rs80958544 | 489077 | 1 | A:G | 0.001 |
| rs81344756 | 592593 | 1.01E-09 | A:G | 0.203 |
| rs319332088 | 638955 | 0.28 | G:A | 0.098 |
| rs331811465 | 643314 | 1 | G:A | 0.001 |
| rs327575709 | 710284 | 1 | A:G | 0.020 |
