# Supplementary figures and images for: Association between MC1R gene and coat color segregation in Shanxia long black pig and Lulai black pig
Source: BMC Genom Data. 2023 Nov 30;24:74. doi: 10.1186/s12863-023-01161-2 (PMC10691012; doi:10.1186/s12863-023-01161-2)

| a |  | b | |  |
| --- | --- | --- | --- | --- |
|  | M 1 2 3 4  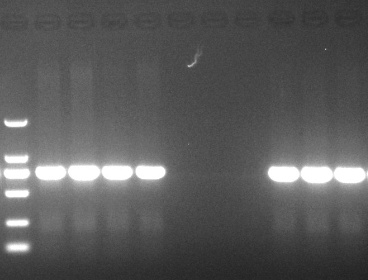  750bp | |  | M 1 2 3 4  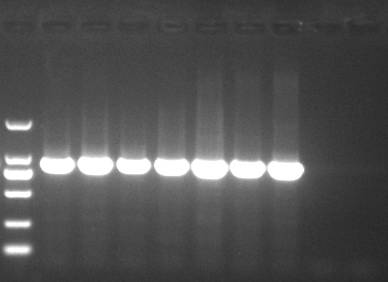  880bp |

**Figure S3 Electropherograms of PCR products.** a. primer 1; b. primer 2.

Supplement: Supplementary file 4 — Supplementary Material 4 [file 12863_2023_1161_MOESM4_ESM.docx]
