# Supplementary material for: Association between MC1R gene and coat color segregation in Shanxia long black pig and Lulai black pig
Source: BMC Genom Data. 2023 Nov 30;24:74. doi: 10.1186/s12863-023-01161-2 (PMC10691012; doi:10.1186/s12863-023-01161-2)
Supplement: Supplementary file 5 — Supplementary Material 5 [file 12863_2023_1161_MOESM5_ESM.docx]

| M 1 2 3 4 5 6 7 8 9 10 11 12  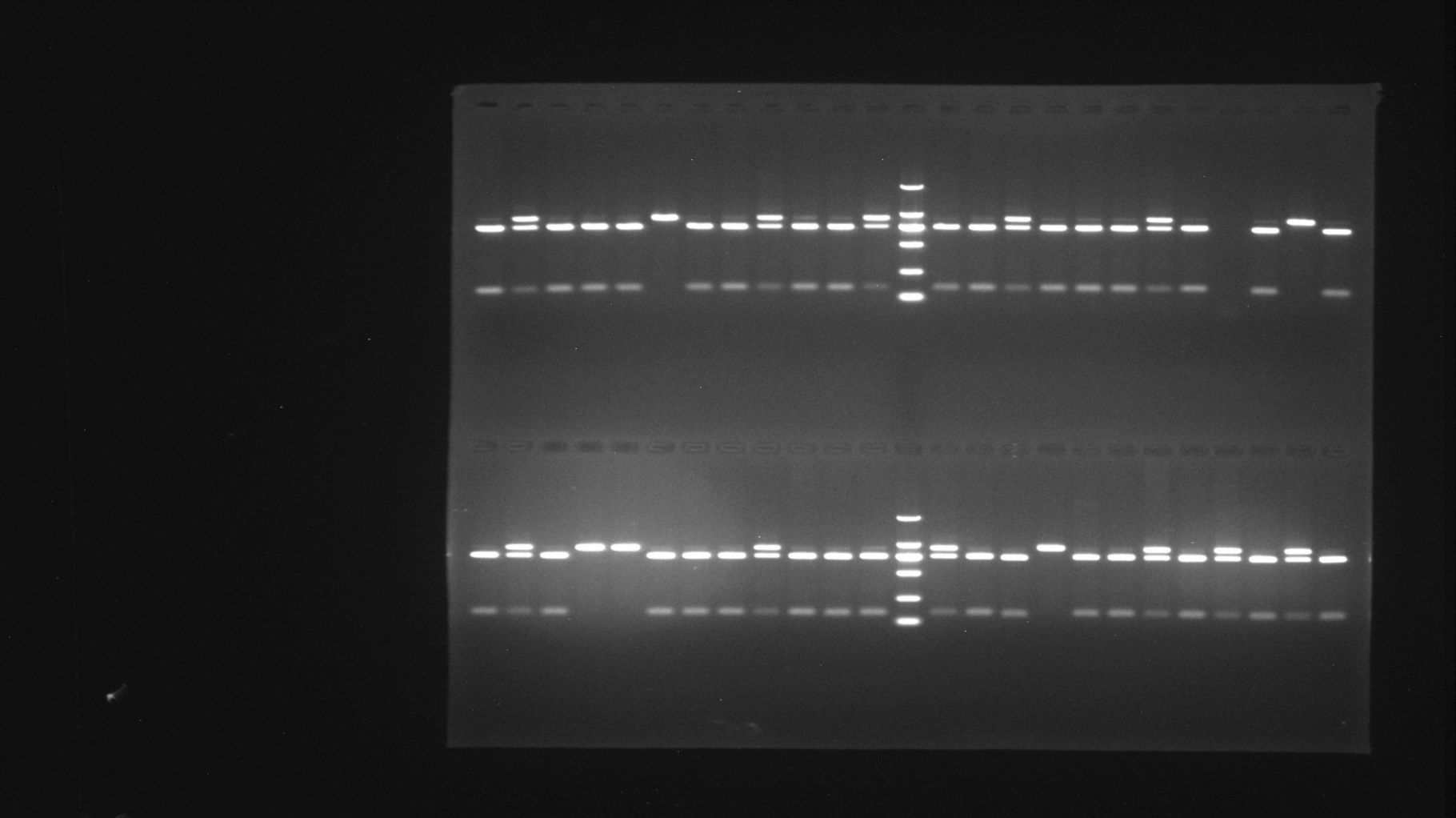  880 bp  750 bp  130 bp |
| --- |

**Figure S4 Electropherogram of PCR-RFLP products for the c.370 G>A locus.** Lane 4 was GG with 1 band; Lanes 1, 7, 9 and 11 were AG with 3 bands; the rest lanes were AA with 2 bands.
