# Supplementary material for: Association between MC1R gene and coat color segregation in Shanxia long black pig and Lulai black pig
Source: BMC Genom Data. 2023 Nov 30;24:74. doi: 10.1186/s12863-023-01161-2 (PMC10691012; doi:10.1186/s12863-023-01161-2)
Supplement: Supplementary file 6 — Supplementary Material 6 [file 12863_2023_1161_MOESM6_ESM.docx]

Table S2 The number of pigs used in each experiment

| Experiment | Population | Black | | Piebald | | Six white | | White limbs | |
| --- | --- | --- | --- | --- | --- | --- | --- | --- | --- |
|  |  | Male | Female | Male | Female | Male | Female | Male | Female |
| Inheritance model test | F_2_ of BL | 14165 | 13852 | 5144 | 5096 | 0 | 0 | 1092 | 1207 |
|  | Licha black pig | 6 | 9 | 4 | 1 | 0 | 0 | 0 | 0 |
| GWAS | BL | 604 | 519 | 16 | 21 | 0 | 0 | 0 | 0 |
| Sanger sequencing | Berkshire | 0 | 0 | 0 | 0 | 9 | 0 | 0 | 0 |
|  | Licha black pig | 0 | 47 | 0 | 0 | 0 | 0 | 0 | 0 |
|  | F_1_ of BL | 1 | 6 | 0 | 0 | 0 | 0 | 0 | 0 |
|  | F_2_ of BL | 12 | 5 | 11 | 14 | 0 | 0 | 5 | 5 |
|  | F_3_ of BL | 2 | 2 | 2 | 1 | 0 | 0 | 2 | 3 |
|  | Lulai black pig | 40 | 3 | 5 | 0 | 0 | 0 | 0 | 0 |
| PCR-RFLP genotyping | Licha black pig | 94 | 0 | 0 | 0 | 0 | 0 | 0 | 0 |
|  | F_1_ of BL | 5 | 20 | 0 | 0 | 0 | 0 | 0 | 0 |
|  | F_2_ of BL | 567 | 65 | 142 | 125 | 0 | 0 | 0 | 0 |
|  | F_3_ of BL | 114 | 114 | 46 | 62 | 0 | 0 | 0 | 0 |
|  | Lulai black pig | 34 | 3 | 0 | 0 | 0 | 0 | 0 | 0 |

Notes: BL, abbreviation for a cross between Berkshire boars and Licha black pig sows.
